# Heat, humidity and health impacts: how causal diagrams can help tell the complex story

**DOI:** 10.1088/1748-9326/ad5a25

**Published:** 2024-07-05

**Authors:** Sidharth Sivaraj, Jakob Zscheischler, Jonathan R Buzan, Olivia Martius, Stefan Brönnimann, Ana M Vicedo-Cabrera

**Affiliations:** 1 Institute of Social and Preventive Medicine, University of Bern, Bern, Switzerland; 2 Oeschger Centre for Climate Change Research, University of Bern, Bern, Switzerland; 3 Department of Compound Environmental Risks, Helmholtz Centre for Environmental Research—UFZ, Leipzig, Germany; 4 Technische Universität Dresden, Dresden, Germany; 5 Physics Institute, University of Bern, Bern, Switzerland; 6 Institute of Geography, University of Bern, Bern, Switzerland

**Keywords:** environmental epidemiology, directed acyclic graphs, wet bulb temperature, compound events

## Abstract

The global health burden associated with exposure to heat is a grave concern and is projected to further increase under climate change. While physiological studies have demonstrated the role of humidity alongside temperature in exacerbating heat stress for humans, epidemiological findings remain conflicted. Understanding the intricate relationships between heat, humidity, and health outcomes is crucial to inform adaptation and drive increased global climate change mitigation efforts. This article introduces ‘directed acyclic graphs’ (DAGs) as causal models to elucidate the analytical complexity in observational epidemiological studies that focus on humid-heat-related health impacts. DAGs are employed to delineate implicit assumptions often overlooked in such studies, depicting humidity as a confounder, mediator, or an effect modifier. We also discuss complexities arising from using composite indices, such as wet-bulb temperature. DAGs representing the health impacts associated with wet-bulb temperature help to understand the limitations in separating the individual effect of humidity from the perceived effect of wet-bulb temperature on health. General examples for regression models corresponding to each of the causal assumptions are also discussed. Our goal is not to prioritize one causal model but to discuss the causal models suitable for representing humid-heat health impacts and highlight the implications of selecting one model over another. We anticipate that the article will pave the way for future quantitative studies on the topic and motivate researchers to explicitly characterize the assumptions underlying their models with DAGs, facilitating accurate interpretations of the findings. This methodology is applicable to similarly complex compound events.

## 1 Introduction

The year 2023 marked the hottest year till date on record, with respect to global mean temperature data going back to 1850 [1]. The emergence of such record-breaking heat anomalies poses significant public health challenges, given the well-established association between heat and mortality, and morbidity burden [2, 3]. A recent study spanning 750 cities across 43 countries found that between 2000 and 2019, there were nearly seven heat-related deaths per 100,000 people per year [4]. Climate change is already playing a substantial role in exacerbating this burden, accounting for nearly 37% of all warm-season heat-related deaths between 1991 and 2018, across multiple countries [5]. While some studies project a slight decrease in non-optimal temperature-related mortality in certain areas and under specific warming scenarios [6–8], owing to a reduction in cold-related mortality, accounting for population aging trends could nullify or even reverse this reduction [9, 10]. Regardless, without adaptation or mitigation measures, heat-related mortality is expected to significantly increase across populations [7, 11]. Therefore, quantification of past and projection of future heat-related health burden is crucial to inform adaptation and drive increased global climate change mitigation efforts.

The accurate quantification of health impacts associated with heat is often challenging in epidemiological research as there are numerous factors and caveats, such as confounding variables, effect modifiers etc., to be accounted for. The role of humidity in heat-related health impacts is one such important, not yet fully resolved question. To delineate the roles of temperature and humidity in heatstress, throughout this article, we use the term ‘heat-related mortality/morbidity’ to exclusively refer to the health impacts associated with temperature exposures higher than the temperature at which the population experiences the lowest risk of impact (referred to as minimum mortality/morbidity temperature [MMT]) [3, 4]. And by ‘humidity’, we refer to the moisture content in the ambient air, irrespective of whether it is expressed as absolute humidity (total mass of water vapour in a given volume of air), specific humidity (total mass of water vapour per unit mass of air), or relative humidity (RH) (percentage of water vapour partial pressure in the air to the saturation water vapour pressure at the same temperature) [12], unless otherwise specified.

In a recent commentary, Baldwin *et al* [13] highlighted how epidemiological studies deal with humidity in heat-related health impact research. They hypothesised that issues in epidemiological analyses, limitations in health and weather data, and physiological factors that might restrict the impact of humidity during real heat waves, as possible reasons for the divergence between physiological research and epidemiological studies around humidity. This manuscript aims to shed light on the issue of analytical complexity in epidemiological research pertaining to humid-heat and health, from a conceptual perspective, by introducing ‘directed acyclic graphs’ (DAGs) as causal models [14, 15]. The goal is to systematically characterise, albeit in a simplified manner, the complex nature of potential associations between heat, humidity, health outcomes and other relevant climate variables using DAGs. While we provide conceptual examples for the modelling premise pertaining to each illustrative DAG, further quantitative assessments with real and simulated data are invited. We believe that this conceptual overview will aid researchers in systematically addressing the complexity pertaining to the problem, and encourage more studies aimed at explicitly addressing current knowledge gaps.

## 2 Current state of research

According to physiological studies, health impacts of heat stress are not restricted to specific levels of temperature alone. Humidity has been shown to play a crucial role in how heat affects the human body [16, 17]. The mechanisms by which heat impacts human health are complex. In brief, the human body has its own cooling system that allows its core temperature to remain constant around a safe range of 36.8 °C ± 0.5 °C, regardless of changes in ambient conditions [18]. Heat stress is the physiological response to increasing heat load on humans, whether that load is environmentally and/or metabolically derived [19]. When heat is not efficiently dissipated, core temperatures will rise, causing heat strain. This is manifested by dangerous physiological responses and pathways, potentially leading to severe organ damage and eventually to death [20, 21]. Temperature and humidity are considered to be the most important contributors to heat stress, as the body cannot release excess heat through evaporation of sweat during hot and humid conditions [20]. Another important factor is solar radiation, a potential confounder associated with both exposure and outcome. Wind also plays an important role through displacement of humid air with less saturated air and thus facilitating evaporative cooling.

Based on this physiological understanding, sub-sequently, numerous combined indices including temperature and humidity were developed over the past century [22] for use in specific disciplines (e.g. military, occupational, or sports medicine) under close-to-experimental conditions [23]. These indices range from complex models focused on the non-linear changes in specific humidity to easy-to-compute metrics focused on RH, to characterize dangerous weather conditions for health in specific population subgroups [24, 25]. One such important metric is the psychrometric or aspirated wet-bulb temperature (*T*_W_). *T*_W_ is the temperature measured using a standard thermometer with its bulb wrapped in a wet cloth exposed to constant airflow and shielded from solar radiation [26, 27]. *T*_W_ can be considered as the lowest temperature attainable through water evaporation alone, under ambient conditions. The natural wet-bulb temperature (T_nwb_), however, is measured with a wet-wick thermometer that sits in a water reservoir unshielded from both solar radiation and wind. *T*_nwb_ thus provide a closer approximation for cooling that can naturally occur through perspiration in the human body [26, 27].

Using a simple energy balance model, Sherwood and Huber [16] proposed that continuous exposure to *T*_W_ of 35 °C for more than four hours represents the upper limit to human survivability. Alarmingly, a global survey of quality-controlled station-based data indicated numerous occurrences of *T*_W_ surpassing 31 °C and 33 °C globally, with two stations already recording multiple daily maximum *T*_W_ values exceeding 35 °C, primarily for durations ranging from 1 to 2 h [28]. These instances of ‘critical’ *T*_W_ exceedances were reported for stations in South Asia, the coastal regions of the Middle East, and coastal areas of southwest North America [28]. In fact, more recent empirical and physiological studies suggest that the ‘critical’ *T*_W_ threshold in reality could be well below the proposed theoretical maximum of 35 °C [17, 29]. This implies that human susceptibility to heat stress may be greater than previously thought. However, physiology-based studies are yet to be sophisticated enough to yield reliable global results, which highlights the importance of epidemiological studies concerning humid-heat.

Environmental epidemiology studies how various physical, biological, and chemical factors in the external environment, when broadly considered, impact health of human populations [30]. While there is a strong consensus regarding the harmful effects of high temperatures on health in environmental epidemiology [31–34], until recently, the health impacts associated with humid-heat had rarely been studied in this field. A limited number of epidemiological studies have focused on examining the distinct impact of humidity on health, and these studies have produced conflicting results. For instance, in a large, multi-location study, Armstrong *et al* [35] identified a minor but protective effect on mortality risk linked to higher humidity levels. Conversely, a separate study in the United States [36] reported a positive association between high humidity and mortality risk for populations. Furthermore, time series regression studies showed that no composite heat stress index (e.g. wet-bulb temperature, heat index etc.) is consistently superior to other indices or dry-bulb temperature in predicting population-level heat-related mortality impacts [37, 38]. The best predictor varied across different age groups, seasons, and cities [37, 38], which may indicate the role of humidity in heat-related health impacts may vary across populations.

The commentary by Baldwin *et al* [13] highlighted the importance of clarifying the role of humidity in heat-related health impacts for robust projection of future climate risks as well as for developing well-informed health adaptation measures. Additionally, we also argue that epidemiological studies provide insights that physiological studies often cannot provide, concerning the generalizability of the findings to different population sub-groups (i.e. children, elderly population, etc.) and assessment of local/regional impacts. In particular, it has been shown that heat-related health impacts are highly variable within and between countries primarily due to differential vulnerability and exposure across populations [39, 40]. Such differences may also exist across populations for humid-heat vulnerability and existing measures (e.g. social, behavioural, institutional etc.) to counter the vulnerability. Therefore, understanding and clarifying the reason(s) behind the lack of consensus between physiological and epidemiological studies is of great significance.

However, the main challenge that epidemiologists face is the inherently complex relationship between heat, humidity, and health due to the multifaceted nature of these factors and their interplay in influencing human well-being. There exists significant variation in the association between temperature and humidity across the planet, which is driven by complex climate drivers, including large-scale circulation patterns, local geographical features, topography, vegetation patterns, soil moisture, oceanic influences etc [41–43]. Moreover, heat and humidity can impact health through various direct and indirect mechanisms, such as heat stress, dehydration, exacerbation of pre-existing health conditions, and the proliferation of vector-borne diseases [44–47]. These factors often interact with socio-economic, environmental, and demographic variables, making it challenging to establish causal relationships and disentangle individual effects of temperature and humidity. Such complexity often means that epidemiological studies either ignore humidity altogether while considering the heat-related health impacts [48, 49] or resort to finding associations between composite heat-stress indices and health impacts [50, 51]. However, neither approach clarifies the exact role played by humidity. Even studies that particularly looked at the role of humidity in heat stress often ‘adjust’ for temperature as a confounder in the regression model without explicitly bringing forth the associated causal diagrams [35].

With DAGs, we aim to explicitly depict the causal assumptions underlying different observational epidemiological study settings that can be used to explore the role of humidity in health outcomes related to heat stress. In such a complex causal network, it is important for researchers to clarify the causal assumptions employed in the models and for readers to incorporate them while interpreting the results. There are numerous precedents even within environmental epidemiology for DAGs being employed to this effect, particularly as an aid for confounder identification [52, 53].

## 3 The causal problem

The question of identifying the role played by humidity in the causal pathway between heat and health impacts is, as the framing suggests, a causal question. Within epidemiology, there exist strong disagreements on the philosophical and methodological foundations for the practice of inferring causation from data [54, 55]. On the one hand, following the ‘causal revolution’ pioneered by Judea Pearl [14] ‘the formal approach to quantitative causal inference’, relies on potential outcomes (i.e. counterfactual reasoning) primarily in interventional study settings [15, 56]. This framework imposes restrictions on the nature of queries to mostly causal effects of manipulable variables, through deliberate human intervention, on the outcome of interest [54, 57]. However, for environmental exposures such as temperature and humidity, such deliberate manipulation of exposures through an interventionist study design is inconceivable at population levels.

On the other hand, historically, epidemiology has relied on a more pluralistic approach to causal inference, that relies on the triangulation of evidence from different sources, and studies with unrelated sources of bias, including clinical, physiological, pathophysiological and observational research [58–60]. The pluralistic viewpoint necessitates consensus between physiological studies and observational studies on the role of humidity in heat-health impacts. Under this view, while there is debate regarding the extent to which DAGs should be incorporated in causal inference, there is still over-whelming consensus that they can be an extremely valuable way of illustrating the context in which causal questions are being asked; in particular, they can illustrate the assumptions being made in analyses, which helps us question the validity of the assumptions [14, 15, 61].

In this article, we do not address the philosophical question of whether observation based studies can provide answers to causal queries regarding the role of humidity in heat-related health impacts. We intend to use DAGs primarily as illustrations of often implicit causal assumptions behind the analytical models employed in observational study settings in environmental epidemiology, which have a significant effect in how the results are interpreted, irrespective of the validity of the results themselves in the larger context of causal inference.

## 4 Causal diagrams: heat, humidity and health

There are many research methodologies employed in environmental epidemiology to characterise the association between exposures (e.g. environmental stressors such as temperature, humidity, pollution, etc.) and responses (e.g. health outcomes such as total mortality, number of hospital admissions, etc.). They include time-series regression studies, case-crossover studies, case-only study designs, cohorts, etc [62]. Irrespective of the methodology, regression models are often employed in such studies to characterise the association of interest. Let

(1) $$\begin{array}{c} Y=\text{Intercept}+\text{f}\left(X;\theta_{X}\right)+\text{f}\left(Z;\theta_{Z}\right)\\ +{\text{residual}}_{X}+{\text{residual}}_{Z} \end{array}$$

be the simplified general form of a regression model representing the association between an outcome *Y* (corresponding to the health impact measure in our case) and exposures *X* (e.g. temperature) and *Z* (e.g. humidity). Here, f(*X; θ*_*X*_) corresponds to the association between the exposure variable *X* and the health outcome Y, characterised by the parameter(s) *θ*_*X*_, given residual_*x*_ and residual_*z*_ are independent. We do not impose any constraints on f to be linear or non-linear.

### 4.1 Confounding and mediation

**Figure 1:**
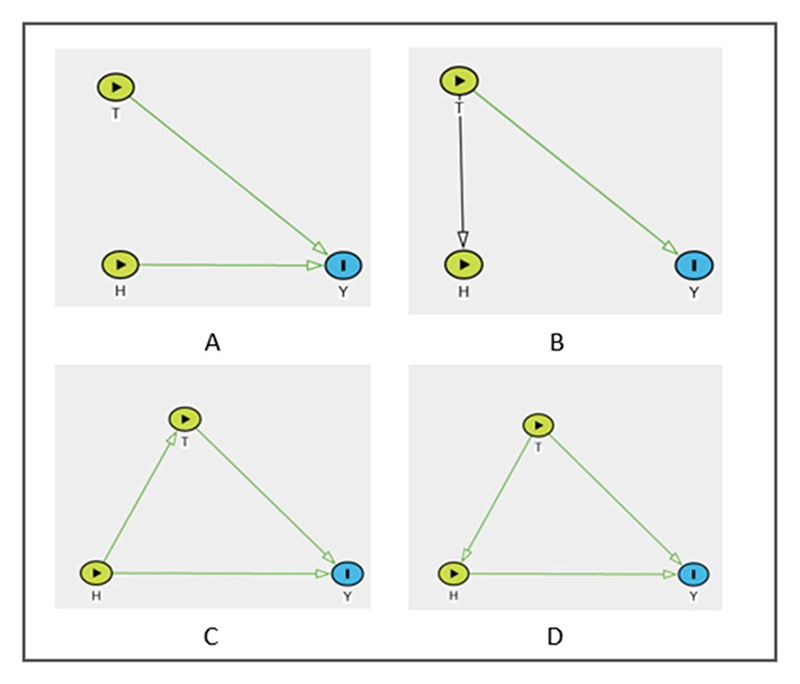
DAGs representing direct causal effects, confounding and mediation. (A) Temperature (T) and humidity (H) have direct causal effects on health outcome (Y). T and H have no causal effects on each other. (B) T has direct causal effects on Y and H. H has no causal effect on Y. (C) H is a confounder of the causal relationship between T and Y. T is a mediator in the causal relationship between H and Y. (D) T is a confounder of the causal relationship between H and Y. H is a mediator in the causal relationship between T and Y.

The DAG in figure 1(A) characterises the assumption that temperature *T* (proxy for heat) and humidity H have causal effects on the health outcome *Y*. i.e. changes in *T* ‘causes’ changes in *Y* if all else is held constant. Also same for *H* and *Y*. These assumptions are indicated by the presence and direction of arrows from *T* and *H* to *Y* in figure 1(A). However there is no relationship between *T* and *H*, indicated by the lack of arrow between *T* and *H*. Under this scenario, one can use the regression models in equations (2) and (3), respectively, to individually assess the association between temperature and health outcome f(*T; θ*_*T*_), and humidity and health outcome f(*H; θ*_*H*_). We omit the residual and the intercept terms from the regression models for ease of representation,

(2) $$Y=\text{f}\left(T;\theta_{T}\right)$$

(3) $$Y=\text{f}\left(H;\theta_{H}\right)$$

Now consider figure 1(B). This DAG corresponds to the assumption that *T* has a causal effect on *H* (not vice-versa) and health outcome *Y*. Moreover, *H* has no causal effect on *Y*. This assumption would also allow one to assess the association between *T* and *Y* using equation (2). Regression model in equation (3) has no physical significance in this scenario as our prior causal model assumes *H* having no causal effect on *Y*.

Whereas the examples above correspond to the scenarios where the individual effects of exposures on the outcome are assessed, the more common practice is to consider humidity as a confounder in the relationship between temperature and health outcomes in epidemiological studies [63–65]. Figure 1(C) corresponds to the causal model underlying this assumption. In this DAG, the arrow from *H* to *T* corresponds to the assumption that changes in *H* ‘causes’ changes in *T*, and not vice-versa (which is indicated by a lack of arrow from *T* to *H*). In addition, we also assume that *H* and *T* have direct causal effects on *Y*. The simplified regression model with this implicit causal assumption assumes the multiple linear regression form of equation (4):

(4) $$Y=f\left(T;\theta_{T}\right)+f\left(H;\theta_{H}\right)$$

Under the prior causal assumption in figure 1(C), from equation (4), we can interpret f(*T; θ*_*T*_) as the magnitude of the association between *T* and *Y* conditioned on the confounder *H*. When we condition, i.e. adjust for humidity in the regression model in equation (4), we eliminate the effect that humidity has on temperature and the health outcome. This can be thought of as creating a hypothetical scenario, i.e. a counterfactual world, where humidity and temperature are considered independent with respect to their effects on health outcomes. It is important to note that under this assumption, it is better to use mass-based measures of humidity, such as specific humidity or absolute humidity, as opposed to RH. Since RH exhibits strong temperature-driven diurnal cycles [25], there is an increased chance of our assumption of lack of arrow from *T* to *H* in figure 1(C) being violated if we use RH. Also, note that *T* mediates the association between *H* and *Y* according to this DAG. Therefore, if we assume the causal model in figure 1(C) prior to analysis, we cannot interpret f(*H; θ*_*H*_) from equation (4) directly as the magnitude of the association between *H* and *Y*. Because by conditioning on *T*, we block part of the effect of *H* on *Y* that is mediated through *T* in this case. Therefore, given the causal model figure 1(C), other analytical techniques such as mediation analysis should be utilised to interpret the total direct (*H* → *Y*) and indirect (*H* → *T* → *Y*) effects of *H* on *Y* separately [66].

However, we can also have a prior causal model, where temperature is the confounder in the association between humidity and health outcome. This is depicted by the DAG in figure 1(D). Here, we assume that the changes in *T* ‘causes’ changes in *H* levels and not vice-versa. Under the causal model figure 1(D), we can directly interpret f(*H; θ*_*H*_) from equation (4) as the magnitude of the association between *H* and *Y* conditioned on the confounder T. Under this causal assumption, in a study spanning multiple countries and cities, Armstrong *et al* [35] showed that the over-all mortality experienced a slight decrease in comparison to the usual mortality following days with elevated RH levels, after accounting for the confounder temperature.

From figures 1(C) and (D), it becomes evident that incorporating both *T* and *H* into a single multivariable regression model, when they are highly correlated with each other, can give rise to multicollinearity issues. Multicollinearity can result in coefficients that are estimated with ambiguity. This may lead to incorrect conclusions, such as humidity (or temperature) does not impact health outcomes [13].

### 4.2 Effect-modification and interaction

In certain studies, researchers explore the role of *H* in modifying the health effects of *T*. This is achieved by incorporating an interaction term that accounts for the combined influence of *T* and *H* in the regression form. While the terms ‘interaction’ and ‘effect modification’ are often used interchangeably, they can hold slightly distinct meanings in the context of causal inference [67]. In practice, effect modification is when we look at how one variable modifies the causal effect of another variable on the outcome. i.e. our interest is in the causal effect of only a single exposure. Whereas with interaction, we are looking for causal effects of both variables on the outcome [68].

**Figure 2:**
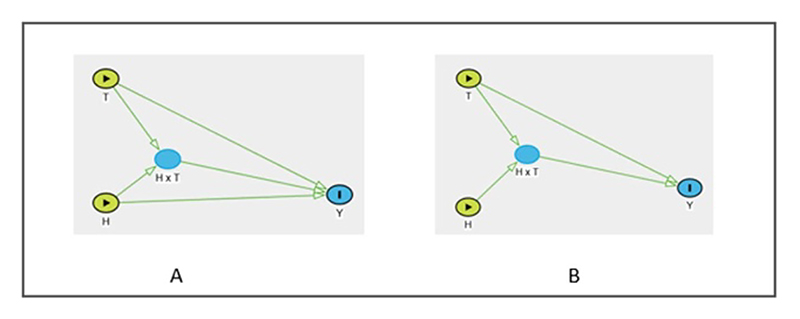
DAGs representing (A) interaction: humidity (*H*) and temperature (*T*) have direct causal effects on health outcome (*Y*). There is an additional interaction effect *H* × *T* on *Y* when both *H* and *T* are present together. (*B*) Effect modification: *T* has direct causal effects on *Y. H* has no direct causal effect on *Y*. Modification of the causal effects of *T* on *Y* by specific exposure levels of *H* is represented by the *H* × *T* node.

Attia *et al* [68] proposed a novel DAG structure that explicitly represents the causal assumptions inherent to interaction and effect modification. The DAG in figure 2(A) depicts the assumption that each of the exposures, i.e. *T* and *H*, has a direct causal effect on *Y*, but it can also be easily understood that when *T* and *H* are present together, there is an additional effect *H* × *T* on *Y*. The assumptions encoded in the DAG is encapsulated in the regression form in equation (5),

(5) $$Y=\text{f}\left(T;\theta_{T}\right)+\text{f}\left(H;\theta_{H}\right)+\text{f}\left(H\times T;\theta_{H\times \text{T}}\right)$$

The DAG in figure 2(B) depicts the causal assumption that *H* ‘modifies’ the association between *T* and *Y*. Here, the lack of an arrow from *H* to *Y* indicates the assumption that *H* has no direct causal effect on *Y*. Moreover, the causal relationship of *H* with *Y* is only important in order to obtain the adjustment sets *j* of the bivariate exposure; {(*H*_*j*_ × *T*)}. With the inclusion of the *H* × *T* node, the direct arrow from *T* to *Y* now represents the average causal effect of *T* for a subpopulation with specific exposure values of *H* and *T* (which is referred to as stratification) [64, 65].

When incorporating an interaction term or stratifying based on humidity and temperature, it is vital to take into account the primary research objective i.e. whether it is focused on causation, description, or prediction, whether the interaction is assumed to be in multiplicative or additive scale and the potential impact on policy decisions [13]. Explicitly distinguishing between interaction and effect modification using DAGs this way will aid the researchers in clarifying their assumptions regarding the role of humidity.

### 4.3 Composite metrics

Many composite indices are designed to assess the effect of humidity on human health in conjunction with other environmental stressors [22]. We intend to highlight the complexity of the causal models associated with using such composite indices in regression models through the example of wetbulb temperature. It also serves as an insightful example of why it becomes difficult to learn about the role of humidity in particular from such an analysis.

**Figure 3:**
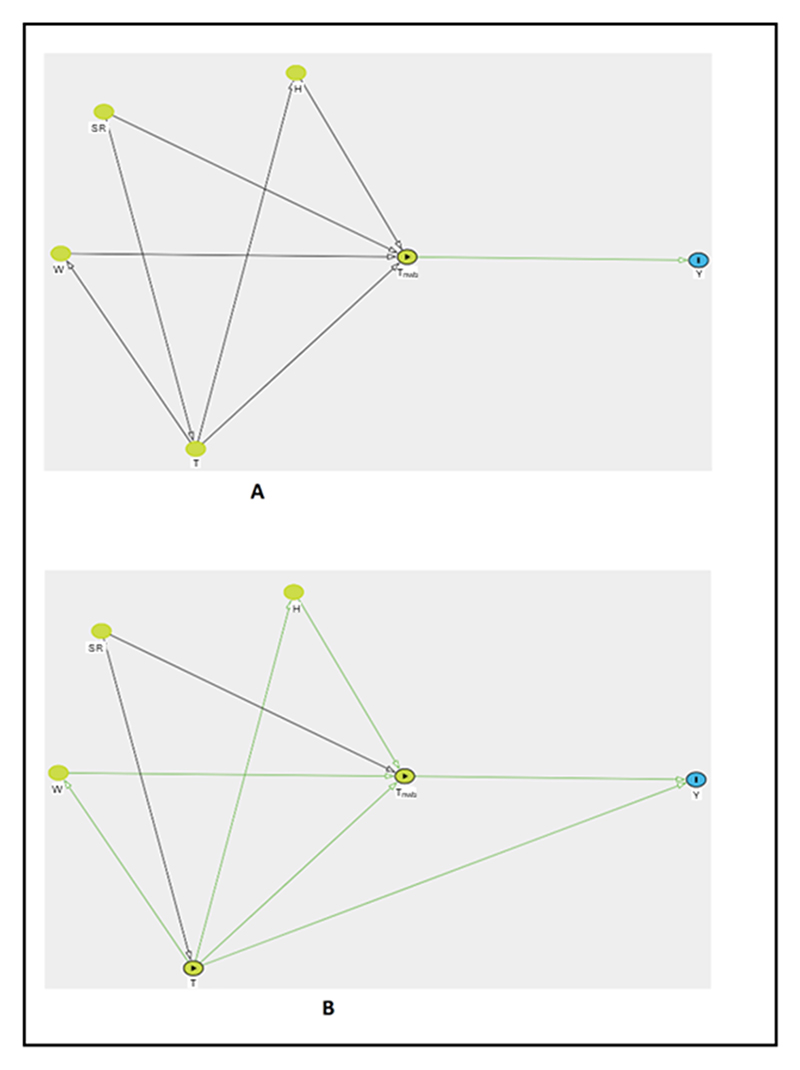
DAGs representing causal assumptions concerning how *T*_nwb_ affects health outcomes (A) relevant causal effects of temperature (*T*), humidity (*H*), wind speed (*W*) and solar radiation (*SR*) on health outcome (*Y*) are completely mediated through the composite index natural wet-bulb temperature (*T*_nwb_). (B) Relevant causal effects of *H* and *W* on *Y* are completely mediated through *T*_nwb_. *T* has direct causal effect on *Y* in addition to causal effect mediated through *T*_nwb_. *SR* has causal effects on *Y* mediated through *T* and *T*_nwb_.

For example, the regression model in equation (6) could correspond to a study analysing the effect of natural wet-bulb temperature (*T*_nwb_) on health outcomes without accounting for any confounding. Here, we make an assumption that *T*_nwb_ measured with a natural wet-bulb thermometer is entirely determined by four variables; temperature (*T*), solar radiation (*SR*), humidity (*H*) and wind speed (*W*). Figure 3(A) could be a causal model justifying the implicit assumption inherent to the model in equation (6),

(6) $$Y=\text{f}\left(T_{\text{nwb}};\theta_{T_{\text{nwb}}}\right)$$

Here in figure 3(A), we interpret the lack of arrows from *T, W, SR* and *H* directly to *Y* as the assumption that all relevant effects of these exposures are mediated through *T*_nwb_ by virtue of how *T*_nwb_ is determined, and no additional individual effects are omitted during such a characterisation. The effects that *SR, T, W* and *H* have on each other are irrelevant under this assumption as we are interested only in the ‘combined’ effect of all the variables on *Y*. However, if one wishes to assess the total indirect effect of *H* on *Y* through mediation analysis in this scenario, it is complicated because *T* confounds the association between *H* and *T*_nwb_. Moreover, *T*_nwb_ is a ‘collider’ [14] in this causal pathway, controlling for which will introduce bias. Therefore, using a composite index mostly limits the possible interpretations to the combined effect of components on the outcome, even under simple causal assumptions. However, in reality, it is too simplistic to assume that *T*_nwb_ could completely mediate all effects that the component variables have on health outcome. Under the assumption that only temperature has some direct causal effect on health outcomes not fully mediated through *T*_nwb_, which is still simplistic, as portrayed by the DAG in figure 3(B), equation (6) can no longer be justified as temperature is a confounder in this new causal model. To overcome this, suppose we condition on temperature, as shown below in equation (7),

(7) $$Y=\text{f}\left(T_{\text{nwb}};\theta_{T_{\text{nwb}}}\right)+\text{f}\left(T;\theta_{T}\right)$$

f(*T*_nwb_; *θ*_*T*nwb_) from the regression model in equation (7) cannot be interpreted as the magnitude of the combined effect of all relevant variables (*SR, T, H* and *W*) on *Y* anymore, under the prior causal model figure 3(B). This is because by conditioning on *T*, we block part of the effect of *T* on *Y* that is mediated through *T*_nwb_ in this case.

**Figure 4:**
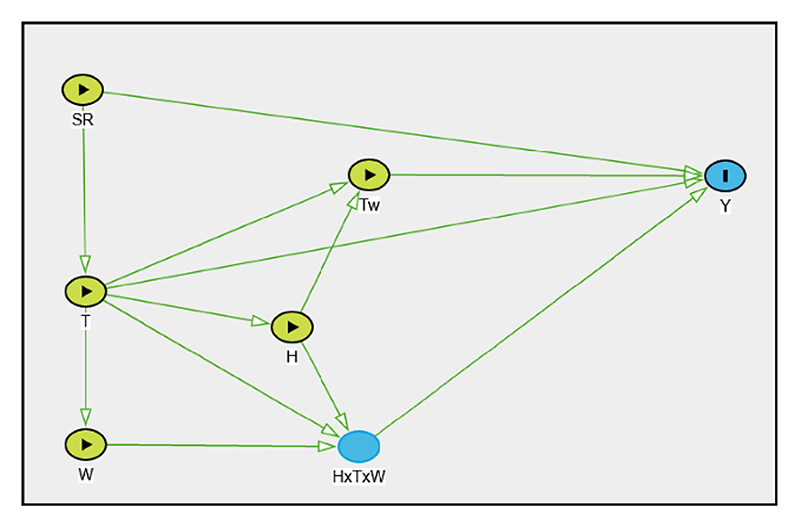
DAG representing causal assumptions concerning psychrometric wet-bulb temperature (*T*_W_). Temperature (*T*) and humidity (*H*) have causal effects on health outcome (*Y*) mediated through *T*_W_. While wind speed (*W*) and solar radiation (*SR*) do not directly influence *T*_W,_ they have potentially relevant causal effects on *Y* through other pathways.

In practice, however, studies tend to use the psychrometric wet-bulb temperature (*T*_W_) to analyse the association between humid-heat and health impacts. This is partly due to the presence of implemented algorithms to compute *T*_W_ from temperature and humidity measurements [69]. Figure 4 represents the potential associations between relevant variables in such a characterisation. From the DAG in figure 4, it is evident that the direct interpretation of f(*T*_W_; *θ*_*T*W_) from an equation such as equation (6) (with *T*_w_ in place of *T*_nwb_) becomes even more muddled. Therefore, the use of composite indices in observational epidemiological studies should be subject to careful consideration and under the strict assumption that the interpretation of the association obtained is strictly constrained on the limitations within which the combined index is constructed.

## 5 Summary

The article highlights the challenges associated with understanding the role of humidity in heat-related health impacts from an environmental epidemiology context. We propose to use DAGs as causal models to clarify the often-implicit causal assumptions made in observational studies, which can significantly affect how results are interpreted.

We have provided causal models in the form of DAGs and the generalised forms of corresponding regression models employed in study settings, considering the roles of humidity as a confounder, mediator, or effect modifier in the relationship between temperature, humidity, and health outcomes (see table 1). The use of composite indices like wet-bulb temperature is also examined, high-lighting the challenges and limitations of interpreting the combined effects of multiple variables on health outcomes.

This conceptual overview deals with the causal assumptions researchers could make, and shape their modelling framework accordingly, while assessing the role of humidity in heat-related health impacts. We are not making comparative statements on the ‘correctness’ of one framework over the other, rather explicitly demonstrating the implications of such choices. Miguel Hernán famously said ‘Draw your assumptions before drawing your conclusions’ [70] regarding the utility of causal models. We believe that the article will contribute to future research by motivating researchers and readers to characterise the assumptions behind models explicitly and to interpret their results accordingly, not limited to the question of the role of humidity on heat-health impacts, but also for other such similarly complex compound events.

## Figures and Tables

**Table 1:** Summary.

Sr. No.	Regression model	Causal model	Conditional independence assumption(s) based on causal model	Association measure(s) based on regression model and causal model
1	*Y* = f (*T*; *θ_T_*)	Figure 1(A)	1) *T* ⊥ *H*	1) f (*T*; *θ_T_*): the association between *T* and *Y*
2	*Y* = f (*T*; *θ_T_*)	Figure 1(B)	1) *Y* ⊥ *H|T*	1) f (*T*; *θ_T_*) : the association between *T* and *Y*
3	*Y* = f (*H*; *θ_H_*)	Figure 1(A)	1) *T* ⊥ *H*	1) f (*H*; *θ_H_*): the association between *H* and *Y*
4	*Y* = f (*T*; *θ_T_*) + f (*H*; *θ_H_*)	Figure 1(C)	None	1) f (*H*; *θ_H_*): not a complete measure 2) f (*T*; *θ_T_*): the association between *T* and *Y* conditioned on H
5	*Y* = f (*T*; *θ_T_*) + f (*H*; *θ_H_*)	Figure 1(D)	None	1) f (*H*; *θ_H_*) : the association between *H* and *Y* conditioned on *T* 2) f (*T*; *θ_T_*): not a complete measure
6	*Y* = f (*T*; *θ_T_*) +f(*H*; *θ_H_*) + f (*H* *×* *T*; *θ_H__×__T_*)	Figure 2(A)	1) *T* ⊥ *H*	1) f (*H*; *θ_H_*): the association between *H* and *Y* conditioned on *T* and additional interaction effect 2) f (*T*; *θ_T_*): the association between *T* and *Y* conditioned on *H* and additional interaction effect 3) f (*H* *×* *T*; *θ_H__×__T_*): the additional interaction effect
7	*Y* = f (*T*; *θ_T_*) +f(*H* *×* *T*; *θ_H__×__T_*)	Figure 2(B)	1) *T* ⊥ *H* 2) *Y* ⊥ *H* *|H* *×* *T*, *T*	1) f (*T*; *θ_T_*): the association between *T* and *Y* conditioned on *H* and effect modification by *H* 2) f (*H* *×* *T*; *θ_H__×__T_*): effect modification by H
8	*Y* = f (*T*_nwb_ ; $\theta_{T_{awb\;}}$	Figure 3(A)	1) *Y ⊥* W | *T*_nwb_ 2) *Y ⊥* *T* | *T*_nwb_ 3) *Y ⊥* *H* *|* *T*_nwb_ 4) *Y ⊥* *SR* | *T*_nwb_ 5) *W* ⊥ *H* | *T* 6) *W* ⊥ *SR* *|T* 7) *H* ⊥ *SR* *|T*	1) f (*T*_nwb_ ; $\theta_{T_{awb\;}}$: the association between *T*_nwb_ and *Y*
9	*Y* = f (*T*_nwb_; $\theta_{T_{awb\;}}$ +f(*T*; *θ_T_*)	Figure 3(B)	1) *Y ⊥ W* | *T*, *T*_nwb_ 2) *Y ⊥ H* | *T*, *T*_nwb_ 3) *Y ⊥ SR* | *T*, *T*_nwb_ 4) *W ⊥ H* | *T* 5) *W ⊥ SR* | *T* 6) *SR ⊥ H* | *T*	1) f (*T*_nwb_ ; *θ*_*T*_nwb__): the association between *T*_nwb_ and *Y* conditioned on *T* (Implying that the causal effect of *T* on *Y* mediated through *T*_nwb_ is blocked) 2) f (*T*; *θ_T_*): not a complete measure

*Y* : health outcome, *T*: temperature, *H*: humidity, *W*: wind velocity, *SR*: solar radiation, *T*_nwb_: natural wet-bulb temperature.

## Data Availability

No new data were created or analysed in this study.
